# Reduction in Primary Operator Radiation Dose Exposure During Coronary Angioplasty Using Radiation Absorbing Drape

**DOI:** 10.7759/cureus.46619

**Published:** 2023-10-07

**Authors:** Amit B Sharma, Richa Agrawal

**Affiliations:** 1 Cardiology, Paras Hospital, Gurugram, IND

**Keywords:** cardiologist, intervention, x-ray, percutaneous coronary intervention, radiation

## Abstract

Background

Percutaneous coronary intervention (PCI) involves the use of ionizing radiation and is a common procedure in a cardiac catheterization suite. The RADPAD® surgical drape (Worldwide Innovations & Technologies, Inc., Lenexa, KS) has been developed to reduce scatter radiation exposure to primary operators during routine PCI procedures. This study aims to assess the efficacy of RADPAD drapes in reducing radiation dose in the catheterization laboratory.

Methods

This was a single-center, prospective, observational study that aimed to compare the primary operator dose received with and without the use of a commercially available shield (RADPAD) during PCI. A total of 53 consecutive patients undergoing PCI were randomized in a 1:1 pattern to receive either the RADPAD (study group) or no RADPAD (control group). Standard shielding and personal protective equipment were used. Radiation exposure to the primary operator, total fluoroscopy time, number of cine acquisitions, and air kerma were recorded for each procedure. A one-way ANOVA calculator, including the Tukey honestly significant difference (HSD) test, was used to compare the mean values of radiation exposure. Scatterplot analysis and linear regression slopes of dose relative to air kerma were performed. All shields were reused once only.

Results

The study compared radiation exposure during PCI procedures between patients who received radiation attenuation devices (RADPAD) and those who did not. The RADPAD group had 30 patients, while the NO RADPAD group had 23 patients. There was a significant difference in the number of coronary angiography and single/multi-vessel PCI procedures between the two groups. There was no significant difference in procedural time or air kerma dose between the groups, but the RADPAD group had a significantly lower radiation dose (mean dose of 3.679 mrem) compared to the NO RADPAD group (mean dose of 8.12 mrem) (p < 0.00001). The primary operator dose per unit of air kerma was also significantly lower in the RADPAD group. Overall, the use of RADPAD resulted in a significant reduction in radiation exposure during PCI procedures.

Conclusion

The present study provides further evidence for the efficacy of using radiation-absorbing drapes (RADPAD) in reducing primary operator radiation dose exposure during coronary angioplasty. The equipment for radiation dose reduction of patients also reduces the radiation dose of medical staff. Therefore, the use of RADPAD is recommended as a safe and effective measure for reducing operator radiation dose exposure during coronary angioplasty.

## Introduction

Percutaneous coronary intervention (PCI) procedures rely on ionizing radiation, which can expose staff members in cardiac catheterization suites to chronic low-dose radiation. This type of radiation is known to cause a small but stochastic risk of inducing malignant disease, skin damage, or eye problems, as reported in multiple studies [1-3]. Cardiovascular imaging and intervention are responsible for almost 40% of the increased exposure, which represents a six-fold increase in radiation exposure [4].

Chronic radiation exposure can have significant impacts on the health and well-being of interventional cardiologists, with several diseases such as brain tumors [5,6], cataracts [7], thyroid disease [8,9], and cardiovascular disease [10] being reported in association with it. To mitigate this risk, the Atomic Energy Regulatory Board (AERB) recommends a whole-body effective dose of 20 mSv/year, averaged over five consecutive years [11].

While radiation exposure is an occupational hazard for interventional cardiologists and other healthcare professionals who work with X-ray-based imaging, it is essential to practice the principle of ALARA (as low as reasonably achievable) in every lab where X-rays and other hazardous radiation are used. The RADPAD® is a sterile surgical drape that contains radiation protection materials (bismuth and barium) developed by Worldwide Innovations & Technologies, Inc. (Lenexa, KS) [12].

The RADPAD is placed between the imaging X-ray beam and the primary operator during routine PCI procedures and other image-guided procedures. The RADPAD is known to reduce scatter radiation exposure to the primary operator [12]. In this study, we aimed to evaluate the effectiveness of RADPAD drapes in reducing the radiation dose experienced by operators during routine PCI procedures, coronary angiography (CAG), chronic total occlusion (CTO), and other image-guided procedures in our catheterization laboratory.

## Materials and methods

Study design

The study was designed as a single-center, prospective, observational study conducted between January 2021 and March 2022. It included 53 consecutive patients undergoing PCI for coronary disease. Patients who underwent angiography only, had previously undergone coronary bypass surgery, or were pregnant were excluded from the study. All patients provided written informed consent prior to participation. The procedures were randomly assigned in a 1:1 ratio to either the "RADPAD" study group or the "NO RADPAD" control group. The study was conducted as part of a quality assurance program aimed at monitoring and reducing the dose to staff in the interventional catheterization laboratory, and local ethics committee approval was obtained (Paras Hospital Ethics Committee; Approval Number: IEC/PH/2020/12/0/3).

Shield

The shield used in this study was a commercially available product called RADPAD, which is lead-free and made of a composite material primarily consisting of bismuth. The manufacturer certifies its shielding properties as being equivalent to 0.125 lead. The shield comes in various sizes and shapes depending on the type of intervention being performed.

Shield placement and reuse

The RADPAD was placed on the patient prior to the start of the procedure, following the manufacturer's standard placement instructions. The ideal placement principle is to position the shield between the primary beam and the primary operator. The RADPAD was reused once only.

Equipment and standard shielding measures

All procedures were performed using a standard Philips FD20 catheterization laboratory (Philips, Amsterdam, Netherlands) and with attention to good radiological practice, such as careful collimation. Standard shielding measures were followed, including a hanging lead curtain beneath the patient table and personal shielding worn by the operator in the form of a lead apron and thyroid collar.

Data collection and analysis

**Figure 1:**
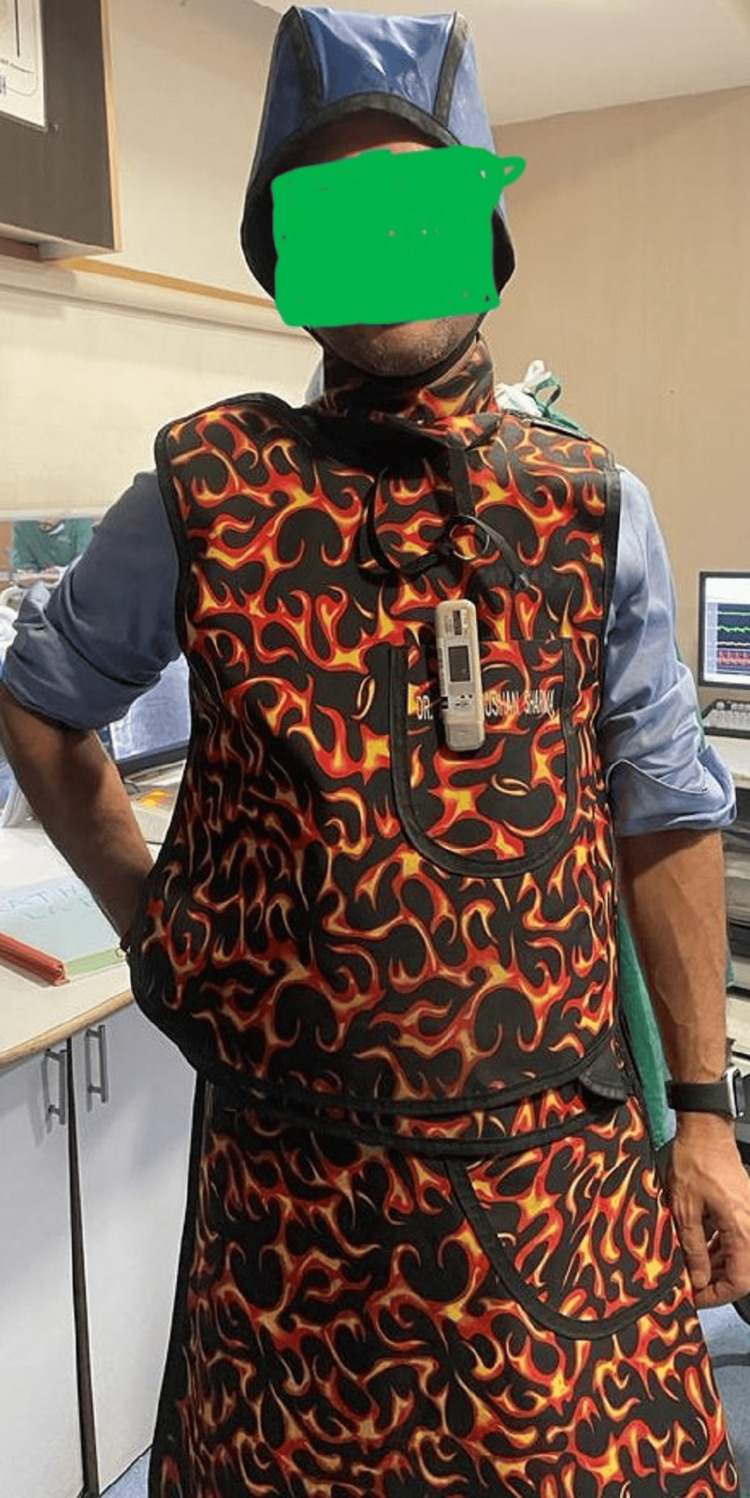
Dosimeter placement on the operator.

Radiation exposure to the primary operator (referred to as the primary operator dose) was measured using an Aloka PDM 127 personal dosimeter (Aloka, Tokyo, Japan) placed on the chest region of the operator (Figure 1), along with the total fluoroscopy time in seconds, the number of cine acquisitions, and the air kerma (AK), which were recorded for each procedure. The primary endpoint of the study was to compare the primary operator received dose relative to the AK for both RADPAD and NO RADPAD cohorts. Continuous variables were reported as mean ± standard deviation or median (range) as appropriate, and categorical variables were reported as frequencies and percentages. All statistical analyses were performed using SPSS version 25.0 (IBM Corp., Armonk, NY). The t-test or Mann-Whitney U test was used to compare continuous variables between the two groups, and the chi-square test or Fisher's exact test was used to compare categorical variables. A p-value < 0.05 was considered statistically significant.

## Results

The RADPAD group had 30 patients, while the NO RADPAD group had 23 patients. The results from Table 1, which presents the demographic profile of the study cohort, showed no significant differences between the RADPAD and NO RADPAD groups in terms of age (mean age: 63.20 ± 11.31 years vs. 61.13 ± 9.92 years, p = 0.367), gender (male: 70.0% vs. 69.6%, p = 0.972), BMI (mean BMI: 27.89 ± 4.46 kg/m² vs. 27.11 ± 5.61 kg/m², p = 0.575), hypertension (73.3% vs. 60.9%, p = 0.335), diabetes mellitus (36.7% vs. 47.8%, p = 0.413), and smoking (36.7% vs. 43.5%, p = 0.615).

**Table 1:** Demographic profile of the study cohort.

Variables	RADPAD (n = 30)	NO RADPAD (n = 23)	P-value
	N (%)	N (%)	
Mean age (years)	63.20 ± 11.31	61.13 ± 9.92	0.367
Gender			
Male (n = 37)	21 (70.0)	16 (69.6)	0.972
Female (n = 16)	9 (30.0)	7 (30.4)	
Mean BMI (kg/m²)	27.89 ± 4.46	27.11 ± 5.61	0.575
Hypertension			
Yes (n = 36)	22 (73.3)	14 (60.9)	0.335
No (n = 17)	8 (26.7)	9 (39.1)	
Diabetes mellitus			
Yes (n = 22)	11 (36.7)	11 (47.8)	0.413
No (n = 31)	19 (63.3)	12 (52.2)	
Smoking			
Yes (n = 21)	11 (36.7)	10 (43.5)	0.615
No (n = 32)	19 (63.3)	13 (56.5)	

Table 2 presents the PCI type and indications among the study cohort. The indications for PCI were stable angina (n = 17), unstable angina (n = 14), non-ST-elevation myocardial infarction (NSTEMI; n = 11), and ST-elevation myocardial infarction (STEMI; n = 13). There were no significant differences between the RADPAD and NO RADPAD groups in terms of indication for PCI (p = 0.992). When looking at the PCI type, the RADPAD group had two CAG procedures and 28 single/multi-vessel PCI procedures, while the NO RADPAD group had nine CAG procedures and 14 single/multi-vessel PCI procedures. This difference was statistically significant (p = 0.003).

**Table 2:** PCI type and indications among study cohort. PCI: percutaneous coronary intervention; NSTEMI: non-ST-elevation myocardial infarction; STEMI: ST-elevation myocardial infarction; CAG: coronary angiography.

Variables	RADPAD (n = 30)	NO RADPAD (n = 23)	P-value
	N (%)	N (%)	
Indication for PCI			
Stable angina (n = 17)	9 (30.0)	7 (30.4)	0.992
Unstable angina (n = 14)	8 (26.7)	8 (34.8)	
NSTEMI (n = 11)	6 (20.0)	5 (21.7)	
STEMI (n = 13)	7 (23.3)	6 (26.1)	
PCI type			
CAG (n = 11)	2 (6.7)	9 (39.1)	0.003
Single/multi-vessel PCI (n = 42)	28 (93.3)	14 (60.9)	

In terms of procedural data, there was no significant difference between the two groups in terms of ST (mean time in minutes for the procedure) and AK (mean air kerma dose). The RADPAD group had a mean ST of 16.55 ± 8.14 and mean AK of 2145.95 ± 1775.98 mGy, while the NO RADPAD group had a mean ST of 13.53 ± 10.95 and mean AK of 1666.16 ± 1506.35 mGy. However, there was a statistically significant difference in dose (mean dose in mrem) between the two groups, with the RADPAD group having a significantly lower mean dose of 3.679 (range: 0.4-14.2) compared to the NO RADPAD group with a mean dose of 8.12 (range: 0.8-41.9) (p < 0.00001). Finally, the PO dose/AK (mean primary operator dose per unit of air kerma) was also significantly lower in the RADPAD group at 0.089 ± 0.078 mrem/minute compared to the NO RADPAD group at 0.234 ± 0.28 mrem/minute (p = 0.009) (Table 3).

**Table 3:** Procedural data of study cohort. ST: standard time for the procedure; AK: air kerma dose; PO dose: primary operator dose.

Procedural variables	RADPAD (n = 30)	NO RADPAD (n = 23)	P-value
ST (min)	16.55 ± 8.14	13.53 ± 10.95	0.254
AK (mGy)	2145.95 ± 1775.98	1666.16 ± 1506.35	0.303
Dose (mrem)	3.68 (0.40-14.2)	8.12 (0.80-41.9)	<0.0001
PO dose/AK (mrem/min)	0.089 ± 0.078	0.234 ± 0.280	0.009

**Figure 2:**
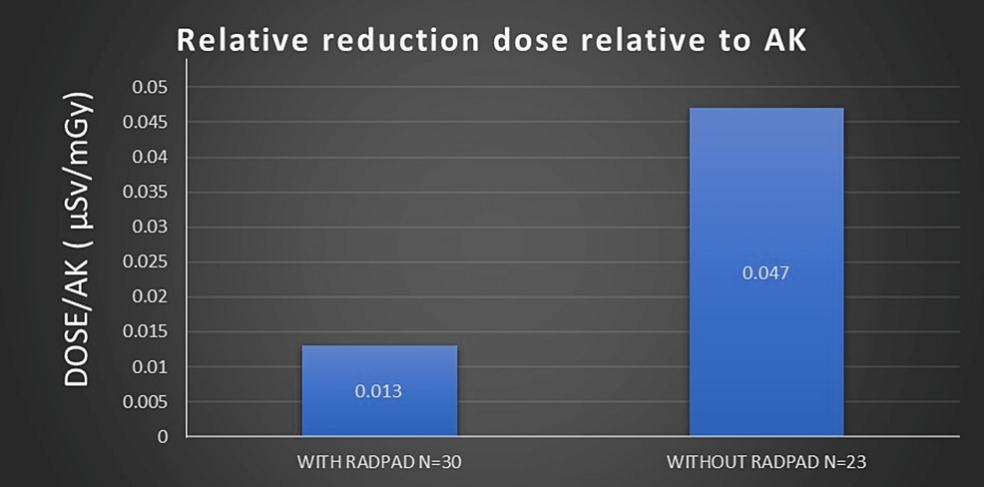
Comparison of relative reduction dose relative to AK among study cohorts. AK: air kerma dose.

Comparing dose relative to AK, the RADPAD cohort received a significantly smaller (72% relative reduction) dose of radiation compared to the NO RADPAD cohort (Figure 2).

## Discussion

The present study investigated the efficacy of using radiation-absorbing drapes (RADPAD) to reduce primary operator radiation dose exposure during coronary angioplasty. The results demonstrated that the use of RADPAD significantly reduced the primary operator's radiation dose exposure compared to the NO RADPAD group. This finding is consistent with previous studies by Mohammadi et al., Vlastra et al., and Iqtidar et al. that have reported that the use of RADPAD significantly reduces radiation exposure during various interventional procedures [13-15].

The significant difference in radiation dose between the RADPAD and NO RADPAD groups in our study suggests that the use of RADPAD can be an effective radiation protection measure. Furthermore, it is noteworthy that the use of RADPAD did not affect procedural outcomes in terms of procedural time (ST) and AK dose. This finding is consistent with previous studies by Ertel et al., Kallinikou et al., Shah et al., Zorzetto et al., and Sciahbasi et al. that have reported that the use of RADPAD does not affect procedural outcomes [16-20].

One interesting finding in our study is the significant difference in the type of PCI between the two groups. The RADPAD group had more single/multi-vessel PCI procedures, while the NO RADPAD group had more CAG procedures. This difference in procedure types may have contributed to the observed difference in radiation dose between the two groups. However, despite this difference in procedure types, the RADPAD group still had a significantly lower radiation dose than the NO RADPAD group.

Another important finding in our study is the significant reduction in primary operator radiation dose exposure relative to air kerma dose (PO dose/AK) in the RADPAD group compared to the NO RADPAD group. This finding suggests that the use of RADPAD can significantly reduce the primary operator's radiation exposure per unit of air kerma dose, which is an important measure of radiation protection. This finding is consistent with previous studies by Gutierrez-Barrios et al., McCutcheon et al., Koukorava et al., Kloeze et al., Balter et al., and Maghbool et al. that have reported a significant reduction in PO dose/AK with the use of RADPAD [21-26].

Limitations

One limitation of our study is the relatively small sample size, which may limit the generalizability of the findings. Additionally, this study did not investigate the long-term effects of radiation exposure, such as the risk of cancer and other radiation-related illnesses, which may require further investigation.

## Conclusions

A novel radiation protection surgical drape offers significant benefits by reducing scatter radiation exposure to staff and operators across a range of PCI procedures. The findings from this study demonstrate a noteworthy 72% reduction in total radiation exposure to primary operators, without any procedure prolongation, when utilizing the RADPAD protection. Therefore, we strongly recommend integrating RADPAD into PCI procedures, despite the additional costs involved. Furthermore, this recommendation extends to non-coronary artery interventions, such as endomyocardial biopsy in patients with cardiomyopathies of heart failure with preserved ejection fraction, regardless of whether the approach is transradial or transfemoral, as well as to electrophysiological ablation procedures.
